# From the Bench to Bedside: Biological and Methodology Considerations for the Future of Companion Diagnostics in Nonsmall Cell Lung Cancer

**DOI:** 10.4061/2011/312346

**Published:** 2011-07-18

**Authors:** Anastasios Dimou, Kevin Harrington, Kostas N. Syrigos

**Affiliations:** ^1^Department of Pathology, Yale University School of Medicine, New Haven, CT 06520-8023, USA; ^2^Section of Cell and Molecular Biology, The Institute of Cancer Research, London SW3 6JB, UK; ^3^3rd Department of Medicine, University of Athens Medical School, 11527 Athens, Greece

## Abstract

Companion diagnostics are an emerging and exciting field in the care of oncology patients. These tests accompany standard diagnostic investigations in cancer patients and function as an aid in treatment decision making. A great number of new compounds are under clinical and laboratory testing in nonsmall cell lung cancer (NSCLC). As the variety of therapeutic options expands in the various settings of the disease, it becomes apparent that specific and sensitive molecular tests are necessary to define the subsets of patients who are going to derive clinical benefit. Testing for epidermal growth factor receptor (EGFR) somatic mutations for the appropriate administration of tyrosine kinase inhibitors is just the beginning. Anaplastic lymphoma kinase (ALK) fusion protein detection and molecular histology classification are promising candidate predictors for clinical benefit from crizotinib and pemetrexed, respectively. This paper summarizes such diagnostics and discusses unanswered questions concerning underlying biology and standardization issues.

## 1. Introduction

Nonsmall cell lung cancer (NSCLC) is the leading cause of cancer-related death worldwide [1]. Adenocarcinoma, squamous cell, and large cell carcinoma are the major histologic types. The majority of patients are diagnosed with metastatic disease and their treatment options are limited to systemically administered modalities often at the cost of significant adverse events. With regard to clinical benefit to toxicity ratio, tailoring treatment to every patient with NSCLC has emerged as a long-term goal. Recent advances in understanding tumor biology have provided new treatment targets as well as exciting insights into designing treatment plans according to unique molecular profiles.

Companion diagnostics are tests that accompany diagnostic investigations in cancer patients and determine whether specific drugs should, or should not, be administered. The history of such tests can be traced back to the estrogen receptor in breast cancer: patients with this disease benefit from antiestrogen treatment if their tumors express the receptor [2, 3]. In addition, patients with breast cancer receive trastuzumab if their tumors express HER2, a transmembrane receptor of the HER family [4]. Other examples include testing for KRAS mutations before prescribing cetuximab treatment in colorectal cancers [5] and testing for the presence of the Philadelphia chromosome for imatinib treatment in chronic and refractory or relapsed acute myeloid leukemias (AMLs) [6]. A companion diagnostic test should be highly reproducible and accurate, as well as rigorously standardized and validated before, it is widely recommended for clinical application.

This paper summarizes recent progress in companion diagnostics in NSCLC. In particular, testing for epidermal growth factor receptor (EGFR) mutations and for anaplastic lymphoma kinase (ALK) fusion proteins is discussed. Those genetic alterations have been linked to response to tyrosine kinase inhibitors (TKIs) and ALK inhibitors, respectively. Furthermore, we comment on assays for accurate and specific histologic classification of NSCLC which are necessary for appropriate use of pemetrexed and bevacizumab treatment.

## 2. EGFR

EGFR is a transmembrane receptor present in the majority of patients with NSCLC [7]. The receptor mediates cellular response to various extracellular signals. It is encoded by the proto-oncogene *EGFR* and demonstrates diverse function in the biology of NSCLC.

EGFR activates two major downstream pathways, mediated by Kirsten rat sarcoma (KRAS) and phosphatidylinositol 3-kinase (PI3K) proteins, respectively. KRAS is a protein with GTPase activity which activates BRAF and finally ERK as part of the mitogen-activated protein kinase (MAPK) signaling cascade. On the other hand, PI3K activates phospholipase C (PLC), protein kinase beta (PKB/AKT), and the mammalian target of rapamycin (mTOR) complex. A set of interactions between these two pathways, as well as positive and negative feedback loops, compose a complicated network which mediates the impact of EGFR and other transmembrane receptors on cell proliferation, inhibition of apoptosis, tumor growth, and invasiveness.

### 2.1. EGFR Mutations

The receptor carries activating mutations in its tyrosine kinase (exons 18–21) domain in a subset of the NSCLC population [8–10]. Patients with certain epidemiologic characteristics (adenocarcinoma histology, never smoking status, female gender, and Asian ethnicity) are more likely to harbor the mutations [8–10]. Most of the mutations are in frame deletions in exon 19 and a point mutation in exon 21 (L858R) [11]. A deletion between codons 746 and 750 accounts for 65–75.5% of the deletions in exon 19 [12].

Erlotinib and gefitinib are compounds that reversibly inhibit the tyrosine kinase activity of EGFR and have been employed in the treatment of patients with NSCLC. Clinical benefit is modest in the unselected population with the disease [13]. However, tumors which harbor activating mutations of the tyrosine kinase domain of EGFR tend to respond to TKIs [8–10]. Patients with the mutations have a prolonged time to progression when treated with gefitinib as a first-line regimen compared to chemotherapy, whereas the opposite is true at the absence of a mutation [14–16]. The results of such clinical trials led to the consensus that an effort should be made to define the mutational status in every newly diagnosed patient with NSCLC in order to decide appropriately on the use of TKIs in the first-line setting [17].

#### 2.1.1. Consensus on EGFR Mutations Testing

The recent clinical trials that have established TKIs treatment in the first-line setting for NSCLC patients with activating EGFR mutations have employed a variety of methods for mutation detection (Table 1). Furthermore, a number of different methods have been described in other studies outside the context of clinical trials [18, 19]. Each of these methods has been validated by comparison to direct sequencing in order to estimate its sensitivity and specificity. However, many of the validation studies have been performed in small cohorts and by a limited number of research groups for each method [20–22]. Taken together, the lack of a common protocol for testing tumors for EGFR mutations and the need for more solid validation of such protocols and procedures in large studies by independent groups indicate the type of studies that will support a consensus on testing for the presence of EGFR mutations. 

The most commonly used mutation detection method is direct DNA sequencing following extraction of DNA from a tumor sample. DNA sequencing is, however, a challenging task when a small biopsy or even a cytology block is the only sample available for companion diagnostics. This is very often the case in the metastatic setting for NSCLC patients. Another limitation is that genotyping methods based on DNA extraction are subject to sample contamination with non-tumor DNA derived from normal or stromal cells. When the percentage of non-tumor to tumor DNA exceeds a certain threshold, it is likely that a mutation might not be detected by direct sequencing as the mutation-specific signal will not surpass the background. Other methods have been developed and reported to have superior sensitivities [23–25]. In addition, the number of malignant cells that need to be present in a sample and the number of different cores or blocks that need to be tested before providing a reliable result on EGFR mutation status are not known. It is largely dependent on the heterogeneity of mutated EGFR expression within a core, across different cores and different tissue blocks. Although EGFR mutations occur early in the tumorigenesis process and one would, therefore, expect them to be homogeneous, tumor heterogeneity of EGFR mutations has not been assessed thoroughly so far. In fact, it has been reported that there is discrepancy in the mutational status between the primary tumors and their metastases [26, 27], as well as a level of heterogeneity within a tissue block [28, 29]. Thus, it is imperative to determine the level of heterogeneity of mutated EGFR in order to propose a standard protocol for reliable mutation detection. 

Besides genotyping, immunohistochemistry (IHC) has become available for the most common mutations [19]. Antibodies have been designed to detect specifically in frame deletions in exon 19 and point mutations in exon 21. Despite the high sensitivity and specificity that was initially reported [12, 19], subsequent studies have shown that the “mutation-specific” antibodies are able to detect only 80% of the full spectrum of mutations [30, 31]. Kitamura et al. [29] have reported sensitivity as low as 47%, probably because of the high frequency of uncommon mutations in their cohort. Most of the studies, however, conclude that deletion-specific antibody methodology is inefficient at detecting deletions in exon 19 other than the “classic” in frame deletion between codons 746 and 750. On the other hand, IHC is available in most pathology laboratories and it is more clinically relevant than genotyping as it assesses mutations at the protein level where a TKI's function occurs. Besides, the assay is highly specific and can be used as an initial assessment before confirmatory genotyping is performed. While still experimental, this in situ method may become a useful research tool in illuminating topics such as the heterogeneity of mutant EGFR and the dynamic range of mutant EGFR expression.

#### 2.1.2. Prediction of Secondary Resistance to TKIs

Even when an EGFR-mutated tumor is initially controlled with EGFR inhibition, resistance emerges typically after a median of 9–12 months [14–16]. Different mechanisms of acquired resistance have been described. About half of the cases become resistant because of a point mutation (T790M) in exon 20 [32, 33] and 20% of the cases because of amplification of hepatocyte growth factor receptor (MET) [34]. MET is a transmembrane receptor which can activate EGFR downstream targets via a parallel pathway. Both resistance mechanisms are present in 10% of the patients who become refractory to tyrosine kinase inhibition after initial response [35]. There is evidence to suggest that both MET amplification and T790M point mutation might be present in a low number of tumor cells before administration of tyrosine kinase inhibitor treatment. These cells are selectively enriched after EGFR inhibition [36, 37]. Recently, it was shown that resistance can emerge as the result of activation of a loop between TGF beta and IL6 [38], or through derepression of FGFR2 and FGFR3 [39] or even through phosphatase and tensin homolog (PTEN) loss [40]. These three additional modes of acquired resistance have not yet been validated in large cohorts of patients and their importance remains to be determined. Taken together, these data strongly support the notion that patients should be tested for the presence of genetic alterations that might cause resistance after failure of TKIs and treated appropriately on the basis of such diagnostics. There are currently drugs that can overcome T790M-mediated resistance as shown in preclinical reports [41]. Efficient MET inhibitors become relevant in cases of MET amplification [42]. Some of these regimens are being tested in ongoing clinical trials. Whether the employment of such modalities should follow the failure of TKIs or, alternatively, should accompany TKIs in the first-line setting in order to prevent the emergence of resistance remains an open question.

### 2.2. Miscellaneous Mutations in the EGFR Pathway

A number of activating mutations have been described in the component molecules of this network among patients with NSCLC. Mutations in KRAS [43] are the most well studied. KRAS mutations cause primary resistance of the tumors to TKIs [43]. However, they are mutually exclusive to EGFR mutations [44] and, at the same time, they are not always present in patients who are resistant to TKIs. Thus, detection of such mutations is inferior to EGFR mutation detection and is not currently recommended as a diagnostic test before EGFR inhibition. In some centers, a strategy of “reflex” testing is followed: patients with adenocarcinoma are tested for the presence of EGFR mutations first and, if negative, they are tested for the presence of KRAS mutations [45] in an effort to obtain information about the driver mutation while saving time, tissue, and resources.

Additional mutations have been identified in numerous other genes [46]. HER2 [47], BRAF [48], and PI3K [49] mutations are found in fewer than 5% of patients [44, 50]. In contrast to KRAS mutations, PI3K mutations can be found in both EGFR mutant or wild-type tumors and, therefore, they might be responsible for primary resistance to TKIs in patients with activating EGFR mutations. However, they are rather rare and their predictive potential has yet to be determined in the clinical setting. As several molecules of the KRAS and PI3K pathways are targets of specific inhibitors tested in ongoing clinical trials, such mutations are likely to become clinically relevant in the future. A convenient assay that combines a multiplex PCR step with a single-base extension sequencing step can reliably test for many different mutations simultaneously and is already employed by some centers [51]. The separation and identification of the different alleles in this assay is performed on the basis of different fluorescence colors and different sizes of allele-specific probes.

**Figure 1:**
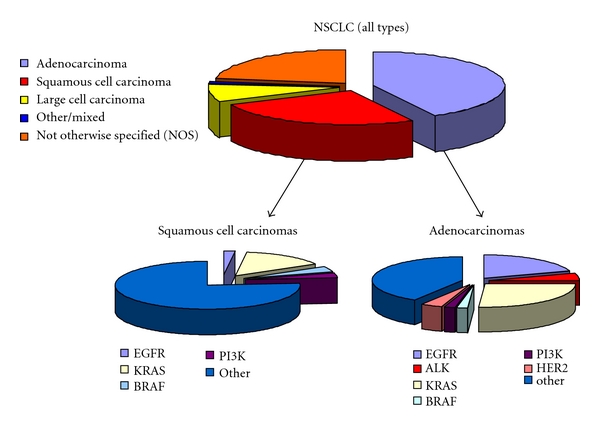
Summary of histotype classification in NSCLC according to the California Cancer Registry [52] and the patterns of mutations seen in squamous cell carcinomas and adenocarcinomas [12, 19, 43, 47, 48, 50].

Figure 1 summarizes the spectrum of histotype classification for NSCLC and the frequency of different mutations in the two major histotypes. It becomes apparent that the “NSCLC puzzle” has yet to become completed, especially in squamous cell carcinomas. Besides, unraveling the puzzle of genetic alterations, certain treatment strategies are needed in conjunction to novel diagnostics.

Since the spectrum of mutations is unique to each histologic type, as shown in Figure 1, it becomes apparent that tumors of different histology are expected to behave differently after exposure to treatments that target specific genetic alterations. In this regard, tyrosine kinase inhibitors are more clinically relevant for the treatment of patients with adenocarcinoma. Likewise, antiangiogenic compounds, like bevacizumab, are contraindicated in patients with squamous cell carcinoma as they might cause lethal bleeding in those patients.

## 3. Anaplastic Lymphoma Kinase (ALK)

Progress in the understanding of EGFR mutations revealed that some tumors rely on single driving genetic alterations, a phenomenon better known as oncogene addiction. The discovery of an “Achilles' heel” in a small subset of the NSCLC population has led to the exciting perspective of exploring novel pathways that are vital to cancer existence and which can potentially be switched off with modern treatment strategies. Reporting of ALK activation in some patients with NSCLC is part of this perspective.

ALK is a transmembrane receptor with tyrosine kinase activity that belongs to the insulin growth factor receptor family, encoded on chromosome 2 (2p23). When bound to its ligand or otherwise activated, it transmits antiapoptotic and cell proliferation signals mediated by KRAS and PI3K pathways [53, 54]. Aberrant activation of ALK was first described in anaplastic large cell lymphoma (ACLC) [54] and in inflammatory myofibroblastic tumor (IMT) [55]. *ALK* contributes to NSCLC biology after being fused with a number of other genes and most frequently Echinoderm microtubule-associated protein 4 (*EML4*) [56, 57]. *EML4* gene is located on chromosome 2 (2p21) and is reversely oriented with *ALK*. *EML4*-*ALK* fusion might occur after a cleavage of the chromosome at a variable site and chromosome inversion, giving rise to different fusion isoforms [58]. EML4-ALK fusion occurs in a mutually exclusive fashion with EGFR or KRAS mutations and, almost exclusively, in adenocarcinomas [59]. However, there have been rare reports of co-existence of EML4-ALK and EGFR mutations [60] or squamous cell carcinoma histology [61]. The presence of EML4-ALK is more likely in patients with certain demographic characteristics, such as never smoking status or younger age [59]. Although a frequency range of 0.4–13.5% [62] has been reported, most studies discover this genetic abnormality at a rate of 2–5% [62–64] in the general population of patients with NSCLC.

### 3.1. Targeting ALK in NSCLC

Tumors that harbor an activated ALK are addicted to the ALK pathway. On the other hand, it appears that these tumors do not respond to EGFR inhibition. Soda et al. showed the oncogenic potential of EML4-ALK in a transgenic mouse model and proved the dependence of the EML4-ALK-positive tumors on the fusion protein [65]. Crizotinib is a dual MET and ALK inhibitor which was already being tested in a clinical trial as a MET inhibitor at the time of ALK discovery. In a phase II trial in 82 patients with activated ALK, crizotinib had a response rate of 57% [66]. A randomized phase III clinical trial in the second-line setting of NSCLC is already recruiting patients with *ALK* fusion gene. Given the highly promising phase II early results, it is likely that testing for the presence of ALK fusion protein will emerge as a diagnostic guide for ALK inhibition. Thus, standardization of this test is an important goal.

### 3.2. Testing for ALK Fusion Gene

A number of different assays have been used for the detection of *ALK* fusion in NSCLC. Fluorescent in situ hybridization (FISH) can be used either as a “fusion” or as a “split-signal” assay [67]. In the fusion variant, different color fluorescence is used for EML4 and for ALK. When a fusion is present, a third color emerges from the overlay. In the split-signal variant, an ALK break-apart probe with different colors (green and red) for the telomeric and the centromeric end of the gene is used. When a fusion is present, 5′ and 3′ probes are split, or an isolated 3′ probe is detected. In the wild-type cells, a merge/yellow signal is obtained. With either variant, a tumor sample is considered to be positive when more than 15% of the tumor cells are positive for the *ALK* fusion gene. Those techniques have proved feasible in a variety of tumor samples like formalin-fixed and paraffin-embedded (FFPE) tissue, pleural effusions, sputum, and small biopsies. FISH has a number of limitations. It requires a level of expertise which is not widely spread in pathology laboratories. In addition, a positive signal (especially with the most widely used split-signal assay) is often subtle and easily missed. Furthermore, a distinction between neoplastic and nonneoplastic cells can be difficult in the absence of direct histological/immunohistochemical correlation on contiguous slides. Finally, the threshold of 15% is rather arbitrary; indeed, it is not followed in all studies [64].

Reverse transcriptase-polymerase chain reaction (RT-PCR) is another way to look for the fusion protein. Primers that generate an amplicon only when a fusion is present are used [56]. An additional step of sequencing can be added for further validation of the result. A limitation to this assay is that RNA is often degraded in FFPE tissue.

A third option is to look for the fusion at the protein level, which is a goal of high clinical relevance since ALK inhibitors are targeting proteins rather than genes. IHC offers in situ information about protein expression and allows correlation with tumor morphology. Besides, it is widely available because of its low cost and simplicity. A number of different antibody clones for ALK detection have been described so far [68–70]. It appears that ALK1 clone lacks sensitivity due to cases with high background whereas D5F3 clone has a more preferable signal-to-background ratio [69]. Interestingly, Mino-Kenudson et al. propose an objective, quantitative, and automated assay for ALK detection [69]. Subjectivity in IHC interpretation and positivity threshold detection, as well as insufficient antibody validation, are known limitations of IHC [71, 72]. 

Martelli et al. [68] reported the presence of EML4-ALK fusion transcripts detected with RT-PCR in normal lung tissue from NSCLC whose tumors were negative for such transcripts. In addition, they failed to detect any protein in IHC, probably because protein levels were too low to be detected. Interestingly, ALK1 along with other clones was used for the detection of the fusion proteins in IHC. Camidge et al. [73] have discovered ALK fusion genes by FISH in normal lung adjacent to ALK-positive or ALK-negative lung tumors. Taken together, these studies underscore the importance of further research in the biology of ALK protein in lung tumors; the possible presence of *ALK* fusion genes in normal lung might drive the spectrum of adverse events from treatment with crizotinib.

## 4. Assays for Histologic Classification of NSCLC

Histological classification of NSCLC has been reviewed and changed several times in the past decades according to emerging knowledge on the disease. However, the need for specific diagnosis of adenocarcinoma or squamous cell carcinoma, the two major types of NSCLC has become clinically relevant only recently.

Current NSCLC treatment optimization is histology specific. In this context, pemetrexed (thymidylate synthase inhibitor) and bevacizumab (anti-angiogenesis monoclonal antibody) are approved for the treatment of non-squamous cell lung carcinomas [74–76]. Histology classification is traditionally based on morphological criteria. Morphology was able to classify correctly only 54% of preoperative cytological and tissue samples [77]. Ou and Zell showed that the percentage of patients diagnosed with NSCLC and classified as “Not Otherwise Specified” (NOS) was much greater when a cytology specimen was used for diagnosis (39% versus 22.1% overall) in the California Cancer Registry [52]. NOS is a diagnosis that should be strictly limited to small biopsies and cytology specimens [78]. Histological misclassification can happen as pathologists agreed with each other in 71.5% of the cases in a study [79]. The adenocarcinoma-specific agreement rate was 82.9% and the squamous-specific agreement rate was 91.2%.

### 4.1. Immunohistochemistry as an Aid for Histology Classification

Several markers have been shown to be differentially expressed in adenocarcinomas and squamous cell carcinomas of the lung. Among them, mucin and thyroid transcription factor 1 (TTF1) are adenocarcinoma-specific proteins, whereas p63 and cytokeratins 5/6 (CK 5/6) are squamous cell carcinoma specific. These markers were applied in a panel of biopsy samples which were classified as NOS by morphological criteria, and the assay was able to classify 73% of the cases into a histological type. Further comparison with matched surgical resections revealed accuracy of 86% [80]. Since none of these proteins is definitive when tested as a single marker, a combination of them creates patterns that favor specific histological types. Furthermore, a quantitative approach that is based on a weighted algorithm of the expression of five proteins is better at classifying NSCLC NOS into squamous cell carcinoma or adenocarcinoma than TTF1/TP63 staining [81]. Taken together, these studies underscore the value of IHC as a diagnostic that aids correct histological classification and, therefore, the optimization of treatment in NSCLC.

### 4.2. Micro-RNA 205

For the subset of cases which remain unclassified after morphological criteria and IHC diagnostics have been applied, more sophisticated tests are emerging. Micro-RNA 205 is a small RNA molecule that is expressed in squamous cell carcinomas of the lung but not in lung adenocarcinomas. A diagnostic test based on the expression of micro-RNA 205, micro-RNA 21, and snRNA U6 can predict squamous cell carcinoma with a sensitivity of 96% and a specificity of 90% [82]. These findings were validated from a separate study where the micro-RNA-205-based diagnostic was able to predict histology perfectly in a set of poorly differentiated squamous cell carcinomas and adenocarcinomas [83]. In the same study, the diagnostic was able to predict correctly histology in 20 out of 21 prospectively collected biopsies with matched resections. A possible limitation of the assay is that it requires a tumor cellularity of at least 50% in the block. Indeed, micro-RNA 205 expression can be artificially altered by contamination from stromal or normal cells. It would be interesting to see whether assessment of micro-RNA 205 with an in situ assay like in situ hybridization (ISH) with micro-RNA-specific probes can overcome this limitation. It is unknown whether difficult cases that need the aid of sophisticated and expensive tests to be classified correctly respond the same to pemetrexed as clear-cut cases. In this regard, it would be interesting to test whether micro-RNA 205 can predict benefit to those drugs as a companion diagnostic superior to histological type.

## 5. Concluding Remarks

At the dawn of companion diagnostics in NSCLC, a number of different tests establish the role of the pathologist as the prescribing physician. Indeed, such tests guide clinical decisions in a similar fashion to trastuzumab administration on the basis of HER2 positivity or antiestrogen treatment on the basis of estrogen receptor positivity in breast cancer. As more and more tests will be required in every newly diagnosed patient, standardization of the assays as well as further clarification of the biology of the candidate markers, becomes increasingly relevant as legitimate goals in both research and clinical practice. Last, but not least, laboratories that perform these tests should be certified by national or global institutions like Clinical Laboratory Improvement Amendments (CLIA).

##  Conflict of Interests

The authors declare that there is no conflict of interests.

## Figures and Tables

**Table 1:** Different assays for detection of EGFR mutation that have been used before TKI treatment in phase III trials in the first-line setting.

Study	Assay
Maemondo et al. [15]	Peptide nucleic acid-locked nucleic acid PCR clamp
Mitsudomi et al. [16]	Fragment analysis, Cycleave method, direct sequencing, peptide nucleic acid-locked nucleic acid PCR, PCR invader
Mok et al. [14]	Amplification refractory mutation system (ARMS)
